# C-reactive protein and white blood cell are associated with frailty progression: a longitudinal study

**DOI:** 10.1186/s12979-022-00280-1

**Published:** 2022-06-03

**Authors:** Zongxue Cheng, Di He, Jun Li, Qiong Wu, Zuyun Liu, Yimin Zhu

**Affiliations:** 1 Department of Epidemiology & Biostatistics, and Department of Respiratory Diseases of Sir Run Run Shaw Hospital, Zhejiang University School of Medicine, Hangzhou, 310058 Zhejiang China; 2 Center for Clinical Big Data and Analytics, Second Affiliated Hospital and Department of Big Data in Health Science, School of Public Health, Zhejiang University School of Medicine, Hangzhou, 310058 Zhejiang China; 3 Cancer Center, Zhejiang University, Hangzhou, 310058 Zhejiang China

**Keywords:** Frailty, Systemic inflammation, C-reactive protein, White blood cells, Frailty index

## Abstract

**Background:**

Systemic inflammation has been linked to diseases and frailty. However, little is known about the effect of systemic inflammation on frailty progression with a longitudinal study design.

**Objectives:**

This study aimed to investigate the associations of two inflammation indicators, C-reactive protein (CRP) and white blood cell (WBC), with frailty progression.

**Methods:**

This study utilized data from the China Health and Retirement Longitudinal Study 2011–2018 (wave 1-wave 4). Frailty index (FI) was calculated using 40 items from wave 1 to wave 4 (range: 0 to 1). Two systemic inflammation biomarkers, CRP and WBC, were measured at baseline (wave 1) and logs transformed as continuous variables or grouped using quartiles. Linear mixed-effect models were used to analyze the associations of these two biomarkers with the progression of frailty with adjustment for potential confounding factors.

**Results:**

The study enrolled 9111 middle-aged and older participants (52.7% females, mean age 58.8 ± 9.3 years). The median follow-up time was 7.0 years. In a fully adjusted model with further adjustment for baseline FI, higher CRP (β for the interaction with time = 0.239, 95% CI: 0.139 to 0.338) and WBC (β for the interaction with time = 0.425, 95% CI: 0.024 to 0.825) significantly accelerated the rate of increase in the FI during the follow-up period. The associations were more pronounced in younger people (< 60 years) than older people (≥60 years).

**Conclusions:**

Higher CRP and WBC accelerated the progression of frailty, particularly in younger groups (< 60 years). The findings suggest the importance of systemic inflammation for the early identification of people at high risk of rapid progression of frailty.

**Supplementary Information:**

The online version contains supplementary material available at 10.1186/s12979-022-00280-1.

## Introduction

Frailty is a geriatric syndrome due to decreased physiologic reserve and increased vulnerability to stressors [1]. Frailty leads to different adverse health outcomes, including falls, disability, morbidity, and early death, posting great economic burden [2–4]. However, there is no consensus on the standardized definition of frailty [5]. Frailty phenotype and frailty index (FI) are two widely accepted measurements of this symptom complex. Frailty index is a cumulative deficit model proposed by Mitnitski et al. in 2001 using continuous scores consisting of function, cognition, comorbidity, and physical performance measures [6]. As a continuous scale, frailty index has a good sensitivity even at the lower part of the frailty continuum (less severe frailty). This property leads to a wide range of population applications of frailty index, not only to measure frailty in older people, but also in middle-aged people.

Systemic inflammation is considered a key component leading to the development of frailty. Systemic inflammation is associated with adverse health outcomes such as cardiovascular diseases, cancer, diabetes, arthritis, and Alzheimer [7–9]. Those adverse health outcomes have been proposed as the intermediate elements that lead to the frailty [10]. C-reactive protein (CRP) and white blood cell (WBC) are common and easily accessible biomarkers of systemic inflammation in clinical diagnosis. Several cross-sectional studies using the frailty phenotype have suggested that the frail population had higher CRP among older adults [11–14]. Similar associations were also found in WBC [15–17]. The previous studies mainly focused on cross-sectional designs measuring frailty at only one time point; however, frailty is not stable and changes over time. It remains to be explored whether CRP and WBC affect frailty progression. In addition, these studies were conducted on older people and there is no evidence from the middle-aged population. Therefore, it is necessary for a long-term follow-up study with multiple measurements of frailty in a large population including middle-aged individuals.

The China Health and Retirement Longitudinal Study (CHARLS) is longitudinal study with a large population-based sample of middle-aged and older adults. In this study, we used the data to investigate the relationships between two inflammatory indicators (CRP and WBC) and progression of frailty evaluated by FI. We hypothesized that higher levels of systemic inflammation biomarkers (CRP and WBC) associated with accelerated frailty progression.

## Methods

### Study design and population

**Fig. 1:**
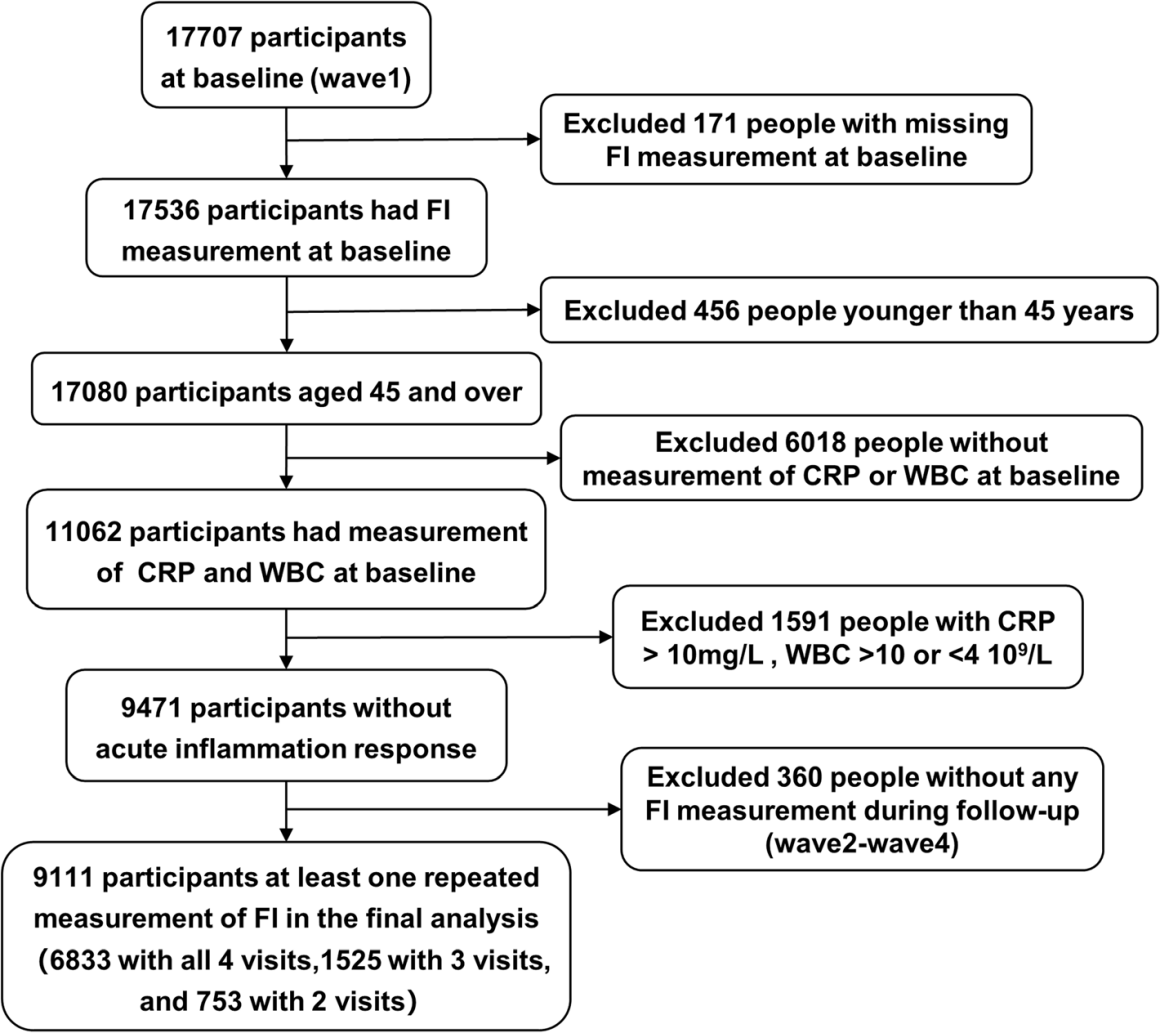
Flowchart of the study population selection

In this study, we used data from wave 1 (2011) to wave 4 (2018) of the CHARLS, which is a large, prospective, national cohort conducted in China. The baseline population was recruited in 2011 from 150 counties/districts in 28 provinces and was followed up every 2 years. The design and methods of this cohort have been described in detail elsewhere [18]. A flow chart of participants selection for the present study was presented in Fig. 1. Approximately 17707 individuals participated in the baseline wave 1 (2011). The participants included in this current study: (i) had completed baseline CRP and WBC data; (ii) had completed baseline FI assessments and at least one follow-up assessment of the FI; (iii) aged 45 years and above. Participants were excluded with acute inflammation response or too low WBC counts, defined as the CRP > 10.00 mg/L or WBC counts > 10 × 10^9^/L, or WBC counts < 4 × 10^9^/L. Finally, a total of 9111 participants were included in the study. There were 6833 participants with all four study visits with FI measured, 1525 participants with three visits, and 753 participants with two visits. And wave 1 (2011) had 9111 participants, wave 2 (2013) had 8534 participants, wave 3 (2015) had 7974 participants and wave 4 (2018) had 7794 participants throughout the longitudinal follow-up.

This study conformed to the provisions of the Declaration of Helsinki. The CHARLS was approved by the Ethics Review Committee of Peking University. Informed consent was obtained from all participants.

### Frailty index

The FI was constructed according to a methodology developed in previous studies [6, 19]. We used 40 health deficits to construct the FI, which ranged across four dimensions: comorbidities, disabilities, cognitive, and depression symptoms. The specific deficit items and cut-off points were presented in Supplemental Table 1. For each participant, the FI was defined as the cumulative sum of the number of present deficits divided by the total number of deficits (*n* = 40). Computed scores ranged from 0 to 1, with higher scores indicated higher degree of frailty. Frailty was defined as a FI ≥ 0.25. The detailed methodology for the construction of the FI was reported in the supplementary materials [see Additional file 1]. Participants were assessed using this FI in wave 1 (2011), wave 2 (2013), wave 3 (2015), and wave 4 (2018).

### C-reactive protein and white blood cell

Venous blood samples were collected after participants fasting overnight at baseline. CRP was measured by immunoturbidimetric assay at the Youanmen Center for Clinical Laboratory of Capital Medical University (CMU) from frozen plasma or whole blood samples. The within-assay coefficient of variation (CV) for CRP was < 1.3%, and between-assay CV was < 5.7%. This CMU laboratory has regular external quality assessments organized by the Chinese Ministry of Health and conducts assay quality control samples daily. White blood cell count analysis was performed on automated analyzers available in the local CDC or town/village health centers laboratories within 141 minutes of collection. These laboratories were involved in routine analytical quality control programs.

### Covariates

Covariates were factors that confound the relationship between indicators of inflammation and frailty in the analysis. We identified potential covariates based on the available empirical clinical evidence [20, 21]. And Supplemental Table 9 described the associations between selected covariates and FI. The covariates involved demographic variables and healthy lifestyles. Demographic variables included age, sex, education levels (illiterate, primary school, middle school or higher), marital status (married or partnered, single), and residence (urban area, rural area). Healthy lifestyles included smoking (ever smokers, never smokers) and drinking (ever drinkers, never drinkers). Body mass index (BMI) was calculated as weight in kilograms divided by square of height in meters.

### Statistical analysis

Continuous variables were expressed as mean (standard deviation) for normal distributions, and median (interquartile range) for skewed distributions. Categorical variables were described as numbers (percentages). Student’s t-test and Chi-square tests were used to compare the statistical difference of continuous variables and categorical variables. In this study, CRP and WBC were logs transformed as continuous variables due to skewed distributions. Meanwhile, CRP and WBC levels were classified into quartiles according to Q1 as the lowest quartile and Q4 as the highest quartile.

To analyze the associations of CRP and WBC at baseline with frailty progression, linear mixed-effect models with a random intercept and slope were performed. Model 1 included CRP (or WBC), follow-up time (in years), the CRP (or WBC) and follow-up time interaction term, age, sex, education levels, marital status, smoking status, drinking status, residence, and BMI. The estimates for CRP (or WBC) reflected the difference of FI by CRP (or WBC) at baseline. The estimate for the time reflected annual changes in the FI. The estimates for CRP (or WBC) by follow-up time interaction term reflected that a unit increment of CRP (or WBC) was associated with a faster or slower rate frailty progression over time in the study. Because baseline FI may influence the progression of frailty, we additionally adjusted for baseline FI in Model 2. To better present the results, all β (95% CIs) were multiplied by 100.

To test whether the association between high inflammation levels and FI progression differed between middle age and old age, a stratified analysis was performed in a sample divided by age 60 years. Subgroup analyses were also conducted stratified by gender. Sensitivity analyses were conducted by excluding frail population at baseline to avoid inverting the causality. Analysis was also performed after the exclusion of the participants with arthritis at baseline. Patients with arthritis will have a reduction in muscle over the course of the disease, which may subsequently result in frailty.

Tests for linear trend were performed by using the median value of each quartile of CRP (or WBC) as a continuous variable in the models.

All analyses were performed using R software (Version 4.0.2). The R package ‘lmerTest’ was used for the linear mixed-effect models. Two-sided *P* values < 0.05 were considered statistically significant.

## Results

### Characteristics of the study population

A total of 9111 participants were included in the study. The mean age at baseline was 58.8 (9.3) years old. A total of 4806 (52.7%) participants were females. The median follow-up times were 7.0 years. The baseline characteristics of the participants were summarized in Table 1 stratified by gender. The medians (interquartile ranges) of CRP (mg/L) and WBC (10^9^/L) were 0.99 (0.54–1.93), 6.00 (5.10–7.10). A total of 1290 participants were defined as frailty, and the prevalence was 14.16%. The median (interquartile range) of the FI was 0.11 (0.05–0.19). Females had a higher FI and a higher prevalence of frailty than males. Women also had lower age, lower education, lower smoking and drinking rates, higher BMI, and lower WBC counts. The baseline characteristics by quartiles of CRP and WBC were correspondingly described in Supplemental Tables 2 and 3. In all participants, CRP quartile categories at baseline were as follows: < 0.54, 0.54–0.98, 0.99–1.92, 1.93–10.00 mg/L; WBC quartile categories at baseline were as follows: 4.0–5.0, 5.1–5.9, 6.0–7.0, 7.1–10.0 10^9^/L.

**Table 1:** Baseline characteristics of participants by gender

Characteristics	Total (*n* = 9111)	Male (*n* = 4305)	Female (*n* = 4806)	*P* value
Age (years), mean (SD)	58.8 (9.3)	59.3 (9.2)	58.3 (9.4)	< 0.001
Education				< 0.001
Illiterate, n (%)	2578 (28.30)	535 (12.43)	2043 (42.51)	
Primary school, n (%)	3704 (40.65)	2001 (46.48)	1703 (35.43)	
Middle school or higher, n (%)	2829 (31.05)	1769 (41.09)	1060 (22.06)	
Married or partnered, n (%)	8076 (88.64)	3943 (91.59)	4133 (86.00)	< 0.001
Ever smokers, n (%)	3586 (39.36)	3215 (74.68)	371 (7.72)	< 0.001
Ever drinkers, n (%)	3564 (39.12)	2865 (66.55)	699 (14.54)	< 0.001
Urban area, n (%)	3256 (35.74)	1513 (35.15)	1743 (36.27)	0.274
BMI (kg/m^2^), mean (SD)	23.52 (3.62)	23.05 (3.34)	23.95 (3.80)	< 0.001
CRP (mg/L), median (IQR)	0.99 (0.54–1.93)	1.00 (0.55–1.93)	0.98 (0.53–1.94)	0.514
WBC (10^9^/L), median (IQR)	6.00 (5.10–7.10)	6.00 (5.20–7.20)	5.92 (5.00–7.00)	< 0.001
Frailty, n (%)	1290 (14.16)	451 (10.48)	839 (17.46)	< 0.001
Frailty index, median (IQR)	0.11 (0.05–0.19)	0.08 (0.04–0.16)	0.12 (0.06–0.21)	< 0.001

Frailty was defined as the frailty index ≥0.25. The age range of total participants was 45–95 years

*P* values were calculated using Student’s t-test for continuous variables and Chi-square tests for categorical variables

*BMI* Body mass index, *CRP* C-reactive protein, *WBC* white blood cell

### Association of CRP with frailty progression

**Fig. 2:**
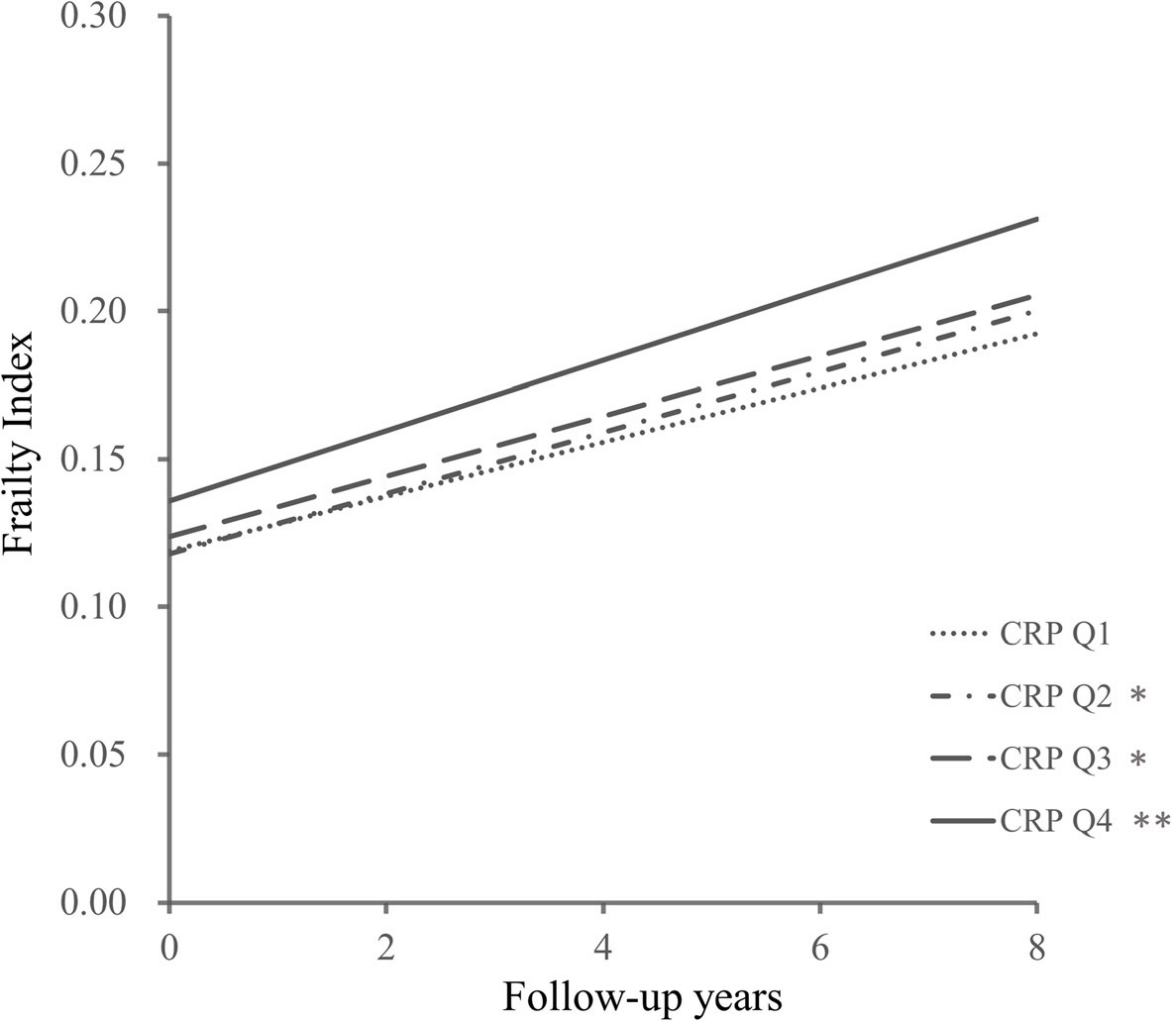
Estimated trajectories of the FI over 7 years by CRP groups using a linear mixed model. Y axis represented the predicted frailty index based on a model adjusted for age, sex, education level, marital status, smoking status, drinking status, residence, and body mass index by CRP quartiles. Different CRP groups were represented using type of lines. represented the group Q4 (the highest CRP group), represented Q3, represented Q2, represented Q1(the lowest CRP group). Participants in the Q4 group had the fastest rate of rise of FI over time, with the Q1 group being the slowest. *P < 0.05, **P < 0.01

As listed in Table 2, participants in the higher CRP were found to have a significant acceleration effect on the FI increase over time, the β for the interaction with time was 0.246 (95% CI: 0.155, 0.338). For quartile groups, compared to the reference group, with increasing interaction term of CRP quartiles by follow-up time, significant dose-response relationships with the FI were observed, with *P* for trend < 0.001. Compared with the lowest quartile of CRP, the β for the interaction with time was 0.109 (95% CI: 0.010, 0.208) in Q2, 0.103 (95% CI: 0.003, 0.202) in Q3, and 0.273 (95% CI: 0.173, 0.373) in Q4. Figure 2 describes the FI trajectories over 7 years by CRP groups, with Q1 as the reference. The accelerating effect of CRP on frailty remained statistically significant with further adjustment for baseline FI in Model 2 (Table 2).

**Table 2:** β (95%CIs) of the associations of C-reactive protein and white blood cell with frailty progression

	CRP		WBC	
	Model1	Model2	Model1	Model2
	β (95% CI)	β (95% CI)	β (95% CI)	β (95% CI)
Q1 × Time	Reference	Reference	Reference	Reference
Q2 × Time	0.109 (0.010, 0.208)	0.112 (0.005, 0.220)	0.023(−0.078, 0.124)	0.019(− 0.091, 0.128)
Q3 × Time	0.103 (0.003, 0.202)	0.097(−0.011, 0.205)	0.037(− 0.065, 0.138)	0.034(− 0.076, 0.143)
Q4 × Time	0.273 (0.173, 0.373)	0.268 (0.160, 0.377)	0.095(−0.006, 0.196)	0.096(−0.014, 0.205)
*P* for Trend	< 0.001	< 0.001	0.057	0.072
Continuous variable ×Time	0.246 (0.155, 0.338)	0.239 (0.139, 0.338)	0.419 (0.049, 0.788)	0.425 (0.024, 0.825)

CRP quartile categories at baseline were as follows: < 0.54, 0.54–0.98, 0.99–1.92, 1.93–10.00 mg/L; WBC quartile categories at baseline were as follows: 4.0–5.0, 5.1–5.9, 6.0–7.0, 7.1–10.0 10^9^/L.

Q1 as the lowest quartile and Q4 as the highest quartile. CRP and WBC as continuous variables were log transformed

β (95% CIs) was calculated by linear mixed-effect models and presented as multiply by 10^2^

Model1: adjusted for age, sex, education level, marital status, smoking status, drinking status, residence, and body mass index; Model 2: additionally adjusted for frailty index at baseline

*CRP* C-reactive protein, *WBC* white blood cell

### Association of WBC with frailty progression

**Fig. 3:**
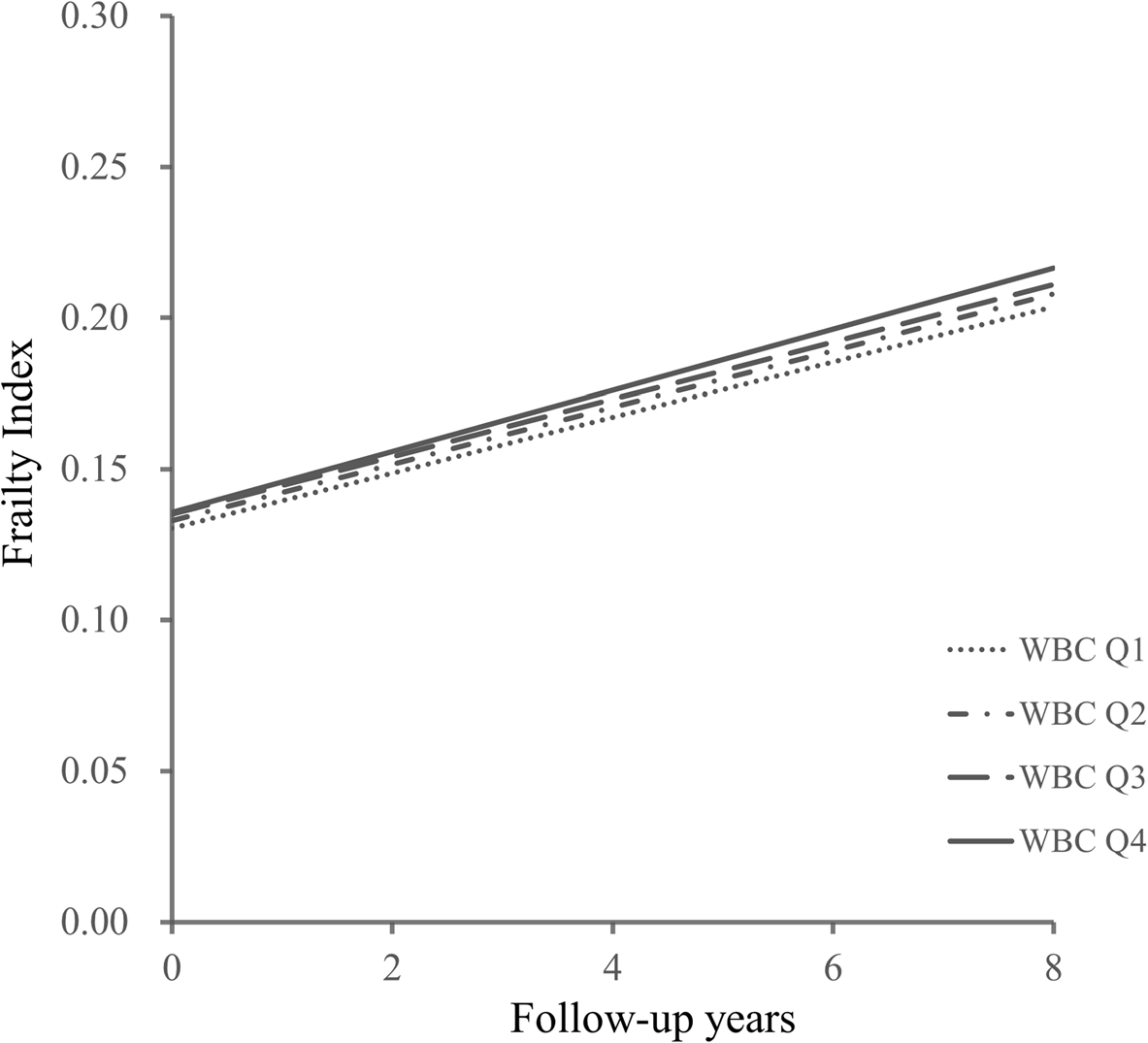
Estimated trajectories of the FI over 7 years by WBC groups using a linear mixed model. Y axis represented the predicted frailty index based on a model adjusted for age, sex, education level, marital status, smoking status, drinking status, residence, and body mass index by WBC quartiles. Different WBC groups were represented using type of lines. represented the group Q4 (the highest WBC group), represented Q3, represented Q2, represented Q1(the lowest WBC group). Participants in the Q4 group had the fastest rate of rise of FI over time, with the Q1 group being the slowest. *P < 0.05, **P < 0.01

Increasing WBC counts at baseline were associated with a significant accelerating effect on the rise of the FI over time, β for the interaction with time was 0.419 (95% CI: 0.049, 0.788). In addition, for quartile groups, Q4 group was close to the significance, with β for the interaction with time was 0.095 (95% CI: − 0.006, 0.196), and the *P* for trend was 0.057. Figure 3 shows the FI trajectories over 7 years by WBC groups, with Q1 as the reference. The accelerating effect of WBC on frailty remained statistically significant with further adjustment for baseline FI in Model 2 (Table 2).

### Association of CRP and WBC with frailty progression by age

As listed in Table 3, higher CRP and WBC had a significant accelerating effect on FI among younger subjects (< 60 years). After further adjustment for baseline FI, β for the interaction with time was 0.239 (95% CI: 0.125, 0.353) for CRP, and 0.729 (95% CI: 0.277, 1.180) for WBC. However, no significance was found in old subjects (≥60 years), β for the interaction with time was 0.096 (95% CI: − 0.080, 0.272) for CRP, and 0.125 (95% CI: − 0.594, 0.843) for WBC.

**Table 3:** β (95%CIs) of the associations of C-reactive protein and white blood cell with frailty progression stratified by age

	CRP		WBC	
	Model1	Model2	Model1	Model2
	β (95% CI)	β (95% CI)	β (95% CI)	β (95% CI)
<60 years (*n* = 5217)				
Q1 × Time	Reference	Reference	Reference	Reference
Q2 × Time	0.020(−0.093, 0.134)	0.025(−0.097, 0.147)	0.032(− 0.084, 0.149)	0.038(− 0.087, 0.163)
Q3 × Time	0.069(− 0.046, 0.183)	0.065(− 0.058, 0.188)	0.086(− 0.030, 0.203)	0.087(− 0.038, 0.212)
Q4 × Time	0.244 (0.129, 0.359)	0.245 (0.121, 0.368)	0.163 (0.048, 0.279)	0.168 (0.044, 0.293)
*P* for Trend	< 0.001	< 0.001	0.003	0.005
Continuous variable ×Time	0.241 (0.135, 0.347)	0.239 (0.125, 0.353)	0.717 (0.297, 1.138)	0.729 (0.277, 1.180)
≥60 years (*n* = 3894)				
Q1 × Time	Reference	Reference	Reference	Reference
Q2 × Time	0.080(−0.094, 0.255)	0.082(− 0.109, 0.274)	0.026(− 0.150, 0.203)	0.012(− 0.181, 0.206)
Q3 × Time	0.043(− 0.132, 0.219)	0.029(− 0.164, 0.222)	−0.004(− 0.181, 0.173)	−0.011(− 0.205, 0.183)
Q4 × Time	0.116(− 0.062, 0.293)	0.101(− 0.093, 0.296)	0.036(− 0.142, 0.214)	0.038(− 0.157, 0.232)
*P* for Trend	0.289	0.423	0.764	0.744
Continuous variable ×Time	0.113(−0.047, 0.274)	0.096(−0.080, 0.272)	0.112(− 0.544, 0.768)	0.125(− 0.594, 0.843)

Q1 as the lowest quartile and Q4 as the highest quartile. CRP and WBC as continuous variables were log transformed

In < 60 years, age range was 45–59 years, and CRP quartile categories at baseline were as follows: < 0.50, 0.50–0.89, 0.90–1.79, 1.80–10.00 mg/L; WBC quartile categories at baseline were as follows: 4.0–5.0, 5.1–5.9, 6.0–7.0, 7.1–10.0 10^9^/L

In ≥60 years, age range was 60–95 years, and CRP quartile categories at baseline were as follows: < 0.61, 0.61–1.10, 1.11–2.15,2.16–10.00 mg/L; WBC quartile categories at baseline were as follows: 4.0–5.0, 5.1–5.9, 6.0–6.9, 7.0–10.0 10^9^/L

β (95% CI) was calculated by linear mixed-effect models and presented as multiply by 10^2^. Model 1: adjusted for age, sex, education level, marital status, smoking status, drinking status, residence, and body mass index; Model 2: additionally adjusted for frailty index at baseline

*CRP* C-reactive protein, *WBC* white blood cell

### Subgroup and sensitivity analysis

Subgroup analysis by sex suggested similar associations in males and females for CRP (Supplemental Table 4). But, the accelerating effect of WBC on frailty was only statistically significant in the female participants (Supplemental Table 4). Sensitivity analysis showed that after excluding frail participants at baseline, the accelerated effect of CRP on frailty remained unchanged compared to the overall participants, while the accelerating effect of WBC was close to the significance (Supplemental Table 5). Among participants without arthritis, the results were consistent with the overall participants (Supplemental Table 6).

## Discussion

In this prospective cohort with a median follow-up time of 7 years, we investigated the association between systemic inflammation markers (CRP and WBC) and frailty progression measured by the FI. To the best of our knowledge, this is the first population-based study to investigate the associations of CRP and WBC with FI change over time. Overall, we observed that higher CRP and WBC were associated with accelerated rate of FI increase, especially in younger adults (< 60 years).

Several previous cross-sectional researches reported a statistically significant relationship between inflammatory markers and FI [22–24]. Using data from a large perspective cohort with multiple repeated FI measurements and a 7-year follow-up, we were able to further explore their longitudinal relationship. Our study contributes to the literature on inflammation and frailty by examining changes in FI trajectories of individuals over time. We found this accelerating effect of CRP and WBC on FI independent of the baseline FI levels. A prospective study showed that the higher CRP on worsening FI, which was confirmed by our findings [25]. However, another longitudinal study conducted among older male individuals, reported no significant association between CRP and incident frailty based on the Fried phenotype [26]. Different measurement tools for frailty are possible explanation, as the Fried phenotype is purely physical, whereas the FI contains more dimensions. The accelerating effect of WBC had a relatively wide confidence interval in our results. This may be because the fact that different subtypes of WBC have different effects on the FI. Neutrophil and monocyte counts were reported to be positively associated with frailty on a cross-sectional basis, while lymphocytes were negatively associated [22, 27, 28]. Due to the lack of data on specific WBC subtypes, we were unable to explore this aspect in more detail. In general, however, the rise in WBC counts is noteworthy.

Moreover, prior researches on this topic conducted on people aged 60 and above. In our study, we expanded the exploration to consider people younger than 60 years of age and found that systemic inflammatory markers were more positively associated with frailty in middle-aged people compared to older people, suggesting that people exposed earlier to high levels of chronic inflammation have a higher risk of frailty. These results proposed the need for the development of relevant screening and intervention programs in the middle-aged population. Nevertheless, the results for the participants aged ≥60 years did not arrive at significance. The findings of the meta-analysis, which included three longitudinal studies and evaluated using frailty phenotype, revealed a similar relationship [13]. The relatively smaller sample size (*n* = 3894) in participants aged ≥60 years may be a likely reason. Notably, older participants had a higher FI compared to the middle-aged, to the extent that there was less space for FI to rise. Furthermore, people who have high levels of inflammatory markers at a younger age may have a genetic susceptibility to many adverse outcomes, leading to a more pronounced effect on frailty progression [29, 30]. In older people, however, there is a phenomenon called ‘Inflammaging’, in which changes in immune reactivity associated with stages of cell differentiation occur with age, with elevated levels of pro-inflammatory factors and the onset of a subclinical, chronic inflammatory state [31]. And high levels of inflammatory markers are still detected in most older adults, even in the absence of clinical diseases and functional impairments [32].

The accelerating frailty progression by inflammation may be due to a number of factors. Inflammation is a risk factor for many age-associated chronic diseases and other adverse health outcomes, including depression, dementia [33–38]. In addition, higher levels of inflammatory markers are linked to a greater loss of muscle strength and mass, which accelerating loss of mobility and physical activity in older people [39, 40]. Meanwhile, all of these are essential constituents for defining frailty. Krisztina Mekli et al. detected associations between two pro-inflammatory gene and the frailty index, and provided genetic evidence for the involvement of the immunological processes in frailty [41]. On the other hand, some researchers have suggested that some of the mechanisms are identical to the pathogenesis of cardiovascular disease. For example, chronic inflammation contributing to frailty through reduced activity and synthesis of a growth factor insulin-like growth factor I that is critical for muscle maintenance and regeneration, and that is protective plaque stability in atherosclerosis [16, 42]. Moreover, inflammation impairs endothelial reactivity and muscle perfusion, interferes with the uptake of long branched chain amino acids, and consequently reduces muscle energy and protein anabolism [43]. Elevated cellular and molecular inflammatory mediators are also inversely related to hemoglobin concentrations, albumin, micronutrients and vitamins.

Our findings also have implications for future clinical practice. Middle-aged individuals with high CRP and WBC levels may be overlooked in terms of risk of frailty, despite measuring levels within the normal range. Therefore, attention should be paid to the individual’s level of inflammation. Measurement of CRP or WBC may help to predict the progression of frailty. These have critical implications for reducing the incidence of frailty and extending healthy life expectancy. However, causality needs to be validated in large clinical trials, and strategies for the use of anti-inflammatory drugs merit further investigation.

There are several strengths in our study. First, we had a relatively large sample size with a prospective design, including a broader age range and a long follow-up period. Second, measurement of frailty index at 4-time points could investigate the association between baseline inflammations and the progression of frailty. Third, we used the appropriate statistical model and adjusted baseline FI. Meanwhile, this study has several limitations. First, the items of frailty index were based on self-reports in this study, such as chronic diseases, which may underestimate the prevalence. Second, the inflammation tests were conducted only once at baseline. The concentrations of these inflammatory markers may change during the follow-up. Third, selection bias may exist due to the exclusion of 350 individuals (3.5%) for lost to follow-up. The results of the non-response analysis suggest that our study participants were healthier than the individuals who were lost to follow-up (Supplemental Table 8). However, even if the lost to follow-up population were not randomly missing, the low attrition rate does not indicate the presence of considerable bias. Additionally, our measurements of inflammatory markers were not comprehensive enough, only CRP and WBC. But other inflammatory markers (e.g., interleukin-6) previously found to be associated with frailty may provide additional information, so this may limit our understanding of the underlying mechanisms of frailty. Finally, although we have adjusted for potential confounders, the presence of unmeasured unknown confounding such as genetic susceptibility may affect our results.

## Conclusion

In summary, this study suggests that elevated CRP and WBC accelerated frailty progression, especially among individuals aged < 60 years. Future studies need to focus on the pathology of frailty. More public health attention is required on inflammation and its adverse consequences to improve quality of life. And the use of inflammation as a biomarker for screening people at high risk of frailty is urgently needed during midlife in the future.

## Supplementary Information

**Additional file 1.**

## Data Availability

The China Health and Retirement Longitudinal Study data are available to registered users at http://charls.pku.edu.cn/zh-CN.

## Abbreviations

BMI: Body mass index
CHARLS: China Health and Retirement Longitudinal Study
CI: Confidence interval
CRP: C-reactive protein
CV: Coefficient of Variation
FI: Frailty index
IQR: Interquartile range
SD: Standard deviation
WBC: White blood cell
