# Supplementary material for: C-reactive protein and white blood cell are associated with frailty progression: a longitudinal study
Source: Immun Ageing. 2022 Jun 3;19:29. doi: 10.1186/s12979-022-00280-1 (PMC9164533; doi:10.1186/s12979-022-00280-1)
Supplement: Supplementary file 1 — Additional file 1. [file 12979_2022_280_MOESM1_ESM.docx]

**Supplementary Methods**

**Construction of the frailty index**

The frailty index was constructed according to a methodology developed in previous studies[[1](#_ENREF_1), [2](#_ENREF_2)]. We included 40 health deficits (Supplementary Table S1), which met the following criteria[[3](#_ENREF_3), [4](#_ENREF_4)]: (1) represent multiple domains of functioning or multiple organ systems; (2) increase in prevalence with age; (3) not be too common before 65 years old (saturate too early); (4) the prevalence should not be less than 1%; (5) the missing rate of each deficit ≤ 5%.

Health deficits included four aspects: comorbidities (items 1-19), disabilities (items 20-38), depression symptoms (item 39), and cognitive (item 40). For each participant, the frailty index was defined as the cumulative sum of the number of present deficits divided by the total number of deficits (n = 40). Computed scores ranged from 0 to 1 continuously, with a higher score indicating a higher degree of frailty. Frailty was defined as the frailty index ≥ 0.25[[5-7](#_ENREF_5)].

For each subject, if more than 30% of the 40 items are missing, they will be excluded. Median imputation for missing data due to the missing rate of each item ≤ 5%.

**References**

1. Searle SD, Mitnitski A, Gahbauer EA, Gill TM, Rockwood K. A standard procedure for creating a frailty index. *BMC Geriatr* 2008,**8**:24.

2. Mitnitski AB, Mogilner AJ, Rockwood K. Accumulation of deficits as a proxy measure of aging. *ScientificWorldJournal* 2001,**1**:323-336.

3. de Vries NM, Staal JB, van Ravensberg CD, Hobbelen JS, Olde Rikkert MG, Nijhuis-van der Sanden MW. Outcome instruments to measure frailty: a systematic review. *Ageing Res Rev* 2011,**10**:104-114.

4. Rockwood K, Mitnitski A. Frailty in relation to the accumulation of deficits. *J Gerontol A Biol Sci Med Sci* 2007,**62**:722-727.

5. Ma L, Chhetri JK, Liu P, Ji T, Zhang L, Tang Z. Epidemiological characteristics and related factors of frailty in older Chinese adults with hypertension: a population-based study. *J Hypertens* 2020,**38**:2192-2197.

6. Song X, Mitnitski A, Rockwood K. Prevalence and 10-year outcomes of frailty in older adults in relation to deficit accumulation. *J Am Geriatr Soc* 2010,**58**:681-687.

7. Kehler DS, Ferguson T, Stammers AN, Bohm C, Arora RC, Duhamel TA*, et al.* Prevalence of frailty in Canadians 18-79 years old in the Canadian Health Measures Survey. *BMC Geriatr* 2017,**17**:28.

**Supplemental Table 1.** Items constituting the Frailty Index and cut-off points.

|  | Items | Coding / Cut-off points |
| --- | --- | --- |
| 1 | Self-reported hypertension | Yes = 1, No = 0 |
| 2 | Self-reported diabetes | Yes = 1, No = 0 |
| 3 | Self-reported chronic lung disease | Yes = 1, No = 0 |
| 4 | Self-reported heart disease | Yes = 1, No = 0 |
| 5 | Self-reported stroke | Yes = 1, No = 0 |
| 6 | Self-reported dyslipidemia | Yes = 1, No = 0 |
| 7 | Self-reported liver disease | Yes = 1, No = 0 |
| 8 | Self-reported cancer | Yes = 1, No = 0 |
| 9 | Self-reported kidney disease | Yes = 1, No = 0 |
| 10 | Self-reported digestive disease | Yes = 1, No = 0 |
| 11 | Self-reported arthritis | Yes = 1, No = 0 |
| 12 | Self-reported asthma | Yes = 1, No = 0 |
| 13 | Self-reported any emotional, nervous, or psychiatric problems | Yes = 1, No = 0 |
| 14 | Self-reported memory-related disease | Yes = 1, No = 0 |
| 15 | Self-reported general health status | Poor or fair = 1, excellent, very good or, good = 0 |
| 16 | Self-reported vision problems | Yes = 1, No = 0 |
| 17 | Self-reported hearing problems | Yes = 1, No = 0 |
| 18 | Self-reported physical Disabilities | Yes = 1, No = 0 |
| 19 | Self-reported brain damage/mental retardation | Yes = 1, No = 0 |
| 20 | BADL: difficulty with dressing | Yes = 1, No = 0 |
| 21 | BADL: difficulty with bathing or showering | Yes = 1, No = 0 |
| 22 | BADL: difficulty with eating | Yes = 1, No = 0 |
| 23 | BADL: difficulty with getting in and out of bed | Yes = 1, No = 0 |
| 24 | BADL: difficulty with using the toilet | Yes = 1, No = 0 |
| 25 | BADL: difficulty with controlling urination and defecation | Yes = 1, No = 0 |
| 26 | IADL: difficulty with managing money | Yes = 1, No = 0 |
| 27 | IADL: difficulty with taking medications | Yes = 1, No = 0 |
| 28 | IADL: difficulty with shopping for groceries | Yes = 1, No = 0 |
| 29 | IADL: difficulty with preparing meals | Yes = 1, No = 0 |
| 30 | IADL: difficulty with doing housework | Yes = 1, No = 0 |
| 31 | Mobility: difficulty with running or jogging about 1 km | Yes = 1, No = 0 |
| 32 | Mobility: difficulty with walking 1 km | Yes = 1, No = 0 |
| 33 | Mobility: difficulty with climbing several flights of stairs without resting | Yes = 1, No = 1 |
| 34 | Mobility: difficulty with getting up from a chair after sitting for long periods | Yes = 1, No = 2 |
| 35 | Mobility: difficulty with stooping, kneeling, or crouching | Yes = 1, No = 3 |
| 36 | Mobility: difficulty with lifting or carrying weights over 10 jins | Yes = 1, No = 4 |
| 37 | Mobility: difficulty with picking up a coin from the table | Yes = 1, No = 5 |
| 38 | Mobility: difficulty with reaching arms above shoulder level | Yes = 1, No = 6 |
| 39 | Depression: CESD-10 | CESD-10 >10 =1, ≤10 =0 |
| 40 | Cognition: (memory test score + orientation test score + serial 7's test + drawing test) / 21 | Continuous, ranging from 0 to 1 |

BADL, basic activities of daily living; IADL, instrumental activities of daily living; CESD-10, Center for Epidemiologic Studies Depression Scale

**Supplemental Table 2.** Baseline characteristics of participants stratified by CRP groups.

| Characteristics | Q1 (n=2229) | Q2 (n=2314) | Q3 (n=2277) | Q4 (n=2291) | *P* value |
| --- | --- | --- | --- | --- | --- |
| Levels of CRP (mg/L) | < 0.54 | 0.54-0.98 | 0.99-1.92 | 1.93-10.00 |  |
| Age (years), mean (SD) | 57.2 (8.9) | 58.6 (9.2) | 59.2 (9.1) | 60.1 (9.6) | <0.001 |
| Male, n (%) | 1027 (46.07) | 1109 (47.93) | 1084 (47.61) | 1085 (47.36) | 0.418 |
| Education |  |  |  |  | 0.104 |
| Illiterate, n (%) | 669 (30.01) | 620 (26.79) | 615 (27.01) | 674 (29.42) |  |
| Primary school, n (%) | 881 (39.52) | 981 (42.39) | 935 (41.06) | 907 (39.59) |  |
| Middle school or higher, n (%) | 679 (30.46) | 713 (30.81) | 727 (31.93) | 710 (30.99) |  |
| Married or partnered, n (%) | 2007 (90.04) | 2074 (89.63) | 2030 (89.15) | 1965 (85.77) | <0.001 |
| Ever smokers, n (%) | 835 (37.46) | 911 (39.37) | 895 (39.31) | 945 (41.25) | 0.079 |
| Ever drinker, n (%) | 885 (39.70) | 936 (40.45) | 866 (38.03) | 877 (38.28) | 0.279 |
| Urban area, n (%) | 681 (30.55) | 812 (35.09) | 873 (38.34) | 890 (38.85) | <0.001 |
| BMI (kg/m²), mean (SD) | 22.48 (2.93) | 23.27 (3.50) | 24.03 (3.59) | 24.30 (4.09) | <0.001 |
| CRP (mg/L), median (IQR) | 0.38 (0.30-0.45) | 0.73 (0.63-0.84) | 1.33 (1.15-1.59) | 3.17 (2.41-4.73) | <0.001 |
| WBC (10^9^/L), median (IQR) | 5.60 (4.90-6.60) | 5.90 (5.10-6.95) | 6.00 (5.19-7.00) | 6.40 (5.48-7.60) | <0.001 |
| Frailty, n (%) | 263 (11.80) | 274 (11.84) | 350 (15.37) | 403 (17.59) | <0.001 |
| Frailty index, median (IQR) | 0.09 (0.04-0.17) | 0.10 (0.04-0.18) | 0.11 (0.05-0.19) | 0.11 (0.06-0.21) | <0.001 |

Frailty was defined as the frailty index ≥ 0.25.

*P* values were calculated using one-way ANOVA for continuous variables and Chi-square tests for categorical variables.

BMI, body mass index; CRP, C-reactive protein; WBC, white blood cell.

**Supplemental Table 3**. Baseline characteristics of participants stratified by WBC groups.

| Characteristics | Q1 (n=2134) | Q2 (n=2335) | Q3 (n=2291) | Q4 (n=2351) | *P* value |
| --- | --- | --- | --- | --- | --- |
| Levels of WBC (10^9^/L) | 4.0-5.0 | 5.1-5.9 | 6.0-7.0 | 7.1-10.0 |  |
| Age (years), mean (SD) | 59.3 (9.3) | 58.9 (9.1) | 58.6 (9.3) | 58.4 (9.4) | 0.007 |
| Male, n (%) | 908 (42.55) | 1140 (48.82) | 1067 (46.57) | 1190 (50.62) | <0.001 |
| Education |  |  |  |  | 0.062 |
| Illiterate, n (%) | 659 (30.88) | 619 (26.51) | 655 (28.59) | 645 (27.44) |  |
| Primary school, n (%) | 831 (38.94) | 967 (41.41) | 931 (40.64) | 975 (41.47) |  |
| Middle school or higher, n (%) | 644 (30.18) | 749 (32.08) | 705 (30.77) | 731 (31.09) |  |
| Married or partnered, n (%) | 1868 (87.54) | 2090 (89.51) | 2039 (89.00) | 2079 (88.43) | 0.193 |
| Ever smokers, n (%) | 714 (33.46) | 931 (39.87) | 896 (39.11) | 1045 (44.45) | <0.001 |
| Ever drinker, n (%) | 794 (37.21) | 964 (41.28) | 869 (37.93) | 937 (39.86) | 0.021 |
| Urban area, n (%) | 754 (35.33) | 878 (37.60) | 820 (35.79) | 804 (34.20) | 0.106 |
| BMI (kg/m²), mean (SD) | 23.21 (3.51) | 23.36 (3.33) | 23.71 (3.78) | 23.79 (3.81) | <0.001 |
| CRP (mg/L), median (IQR) | 0.77 (0.46-1.49) | 0.91 (0.52-1.77) | 1.06 (0.58-2.01) | 1.27 (0.66-2.50) | <0.001 |
| WBC (10^9^/L), mean (SD) | 4.60 (4.30-4.80) | 5.50 (5.30-5.70) | 6.43 (6.20-6.70) | 7.90 (7.50-8.60) | <0.001 |
| Frailty, n (%), median (IQR) | 292 (13.68) | 319 (13.66) | 338 (14.75) | 341 (14.50) | 0.622 |
| Frailty index, median (IQR) | 0.11 (0.05-0.19) | 0.11 (0.04-0.19) | 0.11 (0.05-0.19) | 0.11 (0.05-0.19) | 0.828 |

Frailty was defined as the frailty index ≥ 0.25.

*P* values were calculated using one-way ANOVA for continuous variables and Chi-square tests for categorical variables.

BMI, body mass index; CRP, C-reactive protein; WBC, white blood cell.

**Supplemental Table 4.** Longitudinal associations of C-reactive protein or white blood cell with frailty progression stratified by gender.

|  | CRP | |  | WBC | |
| --- | --- | --- | --- | --- | --- |
|  | Model1 | Model2 |  | Model1 | Model2 |
|  | β (95% CI) | β (95% CI) |  | β (95% CI) | β (95% CI) |
| Males (n=4305) |  |  |  |  |  |
| Q1×Time | Reference | Reference |  | Reference | Reference |
| Q2×Time | 0.081(-0.060, 0.222) | 0.094(-0.060, 0.248) |  | 0.001(-0.145, 0.147) | 0.008(-0.151, 0.168) |
| Q3×Time | 0.067(-0.075, 0.209) | 0.069(-0.086, 0.224) |  | 0.012(-0.131, 0.154) | 0.012(-0.143, 0.168) |
| Q4×Time | 0.270(0.128, 0.412) | 0.281(0.126, 0.436) |  | 0.043(-0.099, 0.186) | 0.052(-0.103, 0.208) |
| *P* for Trend | <0.001 | <0.001 |  | 0.515 | 0.492 |
| Continuous variable ×Time | 0.236(0.104, 0.368) | 0.241(0.097, 0.385) |  | 0.162(-0.368, 0.691) | 0.186(-0.391, 0.764) |
| Females (n=4806) |  |  |  |  |  |
| Q1×Time | Reference | Reference |  | Reference | Reference |
| Q2×Time | 0.140(0.001, 0.279) | 0.139(-0.012, 0.289) |  | 0.019(-0.122, 0.159) | 0.015(-0.137, 0.167) |
| Q3×Time | 0.133(-0.006, 0.272) | 0.122(-0.028, 0.273) |  | 0.065(-0.079, 0.209) | 0.065(-0.091, 0.221) |
| Q4×Time | 0.283(0.144, 0.423) | 0.268(0.117, 0.420) |  | 0.138(-0.004, 0.281) | 0.135(-0.019, 0.289) |
| *P* for Trend | <0.001 | 0.002 |  | 0.038 | 0.057 |
| Continuous variable ×Time | 0.260(0.133, 0.388) | 0.244(0.106, 0.381) |  | 0.666(0.149, 1.183) | 0.655(0.096, 1.213) |

Q1 as the lowest quartile and Q4 as the highest quartile. CRP and WBC as continuous variables were log transformed.

In males, CRP quartile categories at baseline were as follows: <0.55, 0.55-0.99, 1.00-1.92, 1.93-10.00 mg/L; WBC quartile categories at baseline were as follows: 4.0-5.1, 5.2-5.9, 6.0-7.1, 7.2-10.0 10^9^/L.

In females, CRP quartile categories at baseline were as follows: <0.53, 0.53-0.97, 0.98-1.93, 1.94-10.00 mg/L; WBC quartile categories at baseline were as follows: 4.0-4.9, 5.0-5.9, 6.0-6.9, 7.0-10.0 10^9^/L.

β (95% CI) was calculated by linear mixed-effect models and presented as multiply by 10^2^. Model 1 adjusted for age, education level, marital status, smoking status, drinking status, residence, and body mass index; Model 2: additionally adjusted for frailty index at baseline.

CRP C-reactive protein, WBC white blood cell.

**Supplemental Table 5.** Longitudinal associations of C-reactive protein or white blood cell with frailty progression excluding frail participants at baseline.

|  | CRP | |  | WBC | |
| --- | --- | --- | --- | --- | --- |
|  | Model1 | Model2 |  | Model1 | Model2 |
|  | β (95% CI) | β (95% CI) |  | β (95% CI) | β (95% CI) |
| Q1×Time | Reference | Reference |  | Reference | Reference |
| Q2×Time | 0.102(0.001, 0.204) | 0.107(-0.001, 0.216) |  | 0.028(-0.075, 0.131) | 0.029(-0.081, 0.138) |
| Q3×Time | 0.149(0.048, 0.251) | 0.147(0.038, 0.255) |  | 0.060(-0.044, 0.163) | 0.056(-0.054, 0.167) |
| Q4×Time | 0.324(0.222, 0.426) | 0.324(0.215, 0.433) |  | 0.100(-0.003, 0.203) | 0.105(-0.005, 0.214) |
| *P* for Trend | <0.001 | <0.001 |  | 0.045 | 0.050 |
| Continuous variable ×Time | 0.315(0.221, 0.409) | 0.314(0.214, 0.414) |  | 0.373(-0.004, 0.750) | 0.379(-0.023, 0.781) |

Q1 as the lowest quartile and Q4 as the highest quartile. CRP and WBC as continuous variables were log transformed.

In non-frail subjects at baseline, CRP quartile categories at baseline were as follows: <0.53, 0.53-0.94, 0.95-1.86, 1.87-10.00 mg/L; WBC quartile categories at baseline were as follows: 4.0-5.0, 5.1-5.9, 6.0-7.0, 7.1-10.0 10^9^/L.

β (95% CI) was calculated by linear mixed-effect models and presented as multiply by 10^2^.

Model 1: adjusted for age, sex, education level, marital status, smoking status, drinking status, residence, and body mass index; Model 2: additionally adjusted for frailty index at baseline.

CRP C-reactive protein, WBC white blood cell.

**Supplemental Table 6.** Longitudinal associations of C-reactive protein or white blood cell with frailty progression excluding participants with arthritis at baseline.

|  | CRP | |  | WBC | |
| --- | --- | --- | --- | --- | --- |
|  | Model1 | Model2 |  | Model1 | Model2 |
|  | β (95% CI) | β (95% CI) |  | β (95% CI) | β (95% CI) |
| Q1×Time | Reference | Reference |  | Reference | Reference |
| Q2×Time | 0.147(0.028, 0.267) | 0.152(0.023, 0.282) |  | -0.045(-0.167, 0.076) | -0.050(-0.182, 0.082) |
| Q3×Time | 0.080(-0.040, 0.199) | 0.068(-0.062, 0.198) |  | 0.042(-0.080, 0.164) | 0.037(-0.095, 0.169) |
| Q4×Time | 0.277(0.156, 0.397) | 0.280(0.150, 0.411) |  | 0.106(-0.016, 0.227) | 0.112(-0.019, 0.243) |
| *P* for Trend | <0.001 | <0.001 |  | 0.026 | 0.030 |
| Continuous variable ×Time | 0.257(0.147, 0.367) | 0.257(0.137, 0.376) |  | 0.431(-0.013, 0.876) | 0.459(-0.023, 0.940) |

Q1 as the lowest quartile and Q4 as the highest quartile. CRP and WBC as continuous variables were log transformed.

CRP quartile categories at baseline were as follows: <0.53, 0.53-0.97, 0.98-1.90, 1.91-10.00 mg/L; WBC quartile categories at baseline were as follows: 4.0-5.0, 5.1-5.9, 6.0-7.0, 7.1-10.0 10^9^/L.

β (95% CI) was calculated by linear mixed-effect models and presented as multiply by 10^2^.

Model1: adjusted for age, sex, education level, marital status, smoking status, drinking status, residence, and body mass index; Model 2: additionally adjusted for frailty index at baseline.

CRP C-reactive protein, WBC white blood cell.

**Supplemental Table 7.** β (95%CIs) of the associations of C-reactive protein and white blood cell with frailty progression by CRP tertile groups.

|  | CRP | |  | WBC | |
| --- | --- | --- | --- | --- | --- |
|  | Model1 | Model2 |  | Model1 | Model2 |
|  | β (95% CI) | β (95% CI) |  | β (95% CI) | β (95% CI) |
| Q1×Time | Reference | Reference |  | Reference | Reference |
| Q2×Time | 0.104(0.018, 0.190) | 0.106(0.013, 0.200) |  | 0.009(-0.077, 0.096) | 0.006(-0.088, 0.099) |
| Q3×Time | 0.212(0.125, 0.299) | 0.207(0.113, 0.301) |  | 0.088(0.000, 0.175) | 0.089(-0.005, 0.184) |
| *P* for Trend | <0.001 | <0.001 |  | 0.040 | 0.052 |
| Continuous variable ×Time | 0.246(0.155, 0.338) | 0.239(0.139, 0.338) |  | 0.419(0.049, 0.788) | 0.425(0.024, 0.825) |

CRP tertile categories at baseline were as follows: < 0.66, 0.66-1.49, 1.50-10.00 mg/L;

WBC tertile categories at baseline were as follows: 4.0-5.3, 5.4-6.6, 6.7-10.0 10^9^/L.

Q1 as the lowest tertile and Q3 as the highest tertile. CRP and WBC as continuous variables were log transformed.

β (95% CIs) was calculated by linear mixed-effect models and presented as multiply by 10^2^.

Model1: adjusted for age, sex, education level, marital status, smoking status, drinking status, residence, and body mass index; Model 2: additionally adjusted for frailty index at baseline.

CRP C-reactive protein, WBC white blood cell.

**Supplemental Table 8.** Comparison of baseline characteristics between included participants (n=9111) and excluded due to loss to follow-up (n=360)

| Characteristics | Included (n=9111) | Loss to follow-up (n=360) | *P* value |
| --- | --- | --- | --- |
| Age (years), mean (SD) | 58.79 (9.27) | 63.79 (11.54) | <0.001 |
| Education |  |  | 0.011 |
| Illiterate, n (%) | 2578 (28.3) | 128 (35.56) |  |
| Primary school, n (%) | 3704 (40.65) | 131 (36.39) |  |
| Middle school or higher, n (%) | 2829 (31.05) | 101 (28.06) |  |
| Married or partnered, n (%) | 8076 (88.64) | 289 (80.28) | <0.001 |
| Ever smokers, n (%) | 3586 (39.36) | 155 (43.06) | 0.176 |
| Ever drinkers, n (%) | 3564 (39.12) | 142 (39.44) | 0.945 |
| Urban area, n (%) | 3256 (35.74) | 194 (53.89) | <0.001 |
| BMI (kg/m²), mean (SD) | 23.05 (3.34) | 23.95 (3.80) | <0.001 |
| CRP (mg/L), median (IQR) | 0.99 (0.54-1.93) | 1.28 (0.67-2.65) | <0.001 |
| WBC (10^9^/L), median (IQR) | 6.00 (5.10-7.10) | 6.00 (5.24-7.00) | 0.791 |
| Frailty, n (%) | 1290 (14.16) | 112 (31.11) | <0.001 |
| Frailty index, median (IQR) | 0.11 (0.05-0.19) | 0.14 (0.06-0.29) | <0.001 |

Frailty was defined as the frailty index ≥ 0.25.

*P* values were calculated using Student's t test for continuous variables and Chi-square tests for categorical variables.

BMI, body mass index; CRP, C-reactive protein; WBC, white blood cell.

**Supplemental Table 9.** β (95%CIs) and *P* value of the associations of baseline covariates and frailty.

|  | β (95% CI) | *P* value |
| --- | --- | --- |
|  |  |  |
| Education levels |  |  |
| Illiterate | Reference | Reference |
| Primary school | -1.04(-1.62, -0.46) | <0.001 |
| Middle school or higher | -3.47(-4.12, -2.81) | <0.001 |
| Marital status |  |  |
| Married or partnered | Reference | Reference |
| Single | 1.17(0.43, 1.91) | 0.002 |
| Smoking status |  |  |
| Never smoking | Reference | Reference |
| Ever smokers | 1.22(0.59, 1.85) | <0.001 |
| Drinking status |  |  |
| Never drinking | Reference | Reference |
| Ever smokers | 0.20(-0.34, 0.74) | 0.477 |
| Residence |  |  |
| Urban area | Reference | Reference |
| Rural area | 2.23(1.73, 2.72) | <0.001 |
| BMI |  |  |
| Normal | Reference | Reference |
| Overweight | 0.24(-0.29, 0.78) | 0.370 |
| Obesity | 2.46(1.70, 3.23) | <0.001 |
| Underweight | 1.06(0.05, 2.07) | 0.039 |

β (95% CIs) and *P* value were calculated by respective linear regression models which covariate as the independent variable and FI as the dependent variable.

Model adjusted for age and sex.

BMI, body mass index. BMI was grouped by the following ethnically appropriate criteria for Chinese people: underweight is defined as BMI ≥ 28.0 kg/m^2^，normal weight as 18.5 ≤ BMI < 24.0 kg/m^2^, overweight as 24.0 ≤ BMI < 28.0 kg/m^2^ and obesity as BMI ≥ 28.0 kg/m^2^.
